# Peyton's 4-Steps-Approach in comparison: Medium-term effects on learning external chest compression – a pilot study

**DOI:** 10.3205/zma001059

**Published:** 2016-08-15

**Authors:** Tobias Münster, Christoph Stosch, Nina Hindrichs, Jeremy Franklin, Jan Matthes

**Affiliations:** 1 University of Cologne, Cologne interprofessional SkillsLab and Simulation Centre (KISS), Cologne, Germany; 2 University of Cologne, Institute of Medical Statistics, Informatics and Epidemiology (IMSIE), Cologne, Germany; 3 University of Cologne, Department of Pharmacology, Cologne, Germany

**Keywords:** Cardiopulmonary Resuscitation, Education, Peyton's 4 steps approach, Basic Life Support, BLS, CPR, 2 stages, 4 stages, Students, External chest compression

## Abstract

**Introduction: **The external chest compression is a very important skill required to maintain a minimum of circulation during cardiac arrest until further medical procedures can be taken. Peyton’s 4-Steps-Approach is one method of skill training, the four steps being:

1. Demonstration,
2. Deconstruction,
3. Comprehension and
4. Execution.

Based on CPR skill training, this method is widely, allegedly predominantly used, although there are insufficient studies on Peyton’s 4-Steps-Approach for skill training in CPR in comparison with other methods of skill training. In our study, we compared the medium- term effects on learning external chest compression with a CPR training device in three different groups: PEY (Peyton’s 4-Steps-Approach), PMOD (Peyton’s 4-Steps-Approach without Step 3) and STDM, the standard model, according to the widely spread method “see one, do one” (this is equal to Peyton’s step 1 and 3).

**Material and Methods: **This prospective and randomised pilot study took place during the summer semester of 2009 at the SkillsLab and Simulation Centre of the University of Cologne (Kölner interprofessionelles Skills Lab und Simulationszentrum - KISS). The subjects were medical students (2^nd^ and 3^rd^ semester). They volunteered for the study and were randomised in three parallel groups, each receiving one of the teaching methods mentioned above. One week and 5/6 months after the intervention, an objective, structured single assessment was taken. Compression rate, compression depth, correct compressions, and the sum of correct checklist items were recorded. Additionally, we compared cumulative percentages between the groups based on the correct implementation of the resuscitation guidelines during that time.

**Results: **The examined sample consisted of 134 subjects (68% female; age 22±4; PEY: n=62; PMOD: n=31; STDM: n=41). There was no difference between the groups concerning age, gender, pre-existing experience in CPR or time of last CPR course. The only significant difference between the groups was the mean compression rate (bpm): Group 1 (PEY) with 99±17 bpm, Group 2 (PMOD) with 101±16 bpm and Group 3 (STDM) with 90±16 bpm (p=0,007 for Group 3 vs. Group 1 and Group 3 vs. Group 2, Mann-Whitney- U-Test). We observed no significant differences between the groups after the second assessment.

**Conclusion: **Our study showed that there are no essential differences in external chest compression during CPR performed by medical students dependent on the teaching method (Peyton vs. “Non-Peyton”) implemented with regard to the medium-term effects. The absence of benefits could possibly be due to the simplicity of external chest compression.

## Zusammenfassung

**Einführung: **Die extrakorporale Herzdruckmassage ist eine wichtige Fertigkeit, um ein Minimum der Organdurchblutung bei Patienten/-innen mit Herz-Kreislauf-Stillstand zu gewährleisten, bis weitere medizinische Hilfe geleistet werden kann. Eine Methode um diese Fertigkeit zu vermitteln ist der Ansatz nach Peyton. Dieser besteht aus 4 Schritten:

1. Demonstration,
2. Dekonstruktion,
3. Verständnis und
4. Durchführung.

Bezogen auf die kardiopulmonale Reanimation wird eine Überlegenheit dieser Methode gegenüber Anderen angenommen, ist jedoch bisher durch Studien nicht ausreichend gesichert. In unserer Studie haben wir den mittelfristigen Lernerfolg durch die 4-Schritt-Methode nach Peyton („PEY“) mit dem der Modifikation „PMOD“ (4-Schritt-Methode ohne Schritt 3) und dem „STDM“ (Standardmodell, entsprechend der weit verbreiteten Methode „See one Do one“, entspricht der 4-Schritt-Methode ohne Schritt 1 und 3,) anhand von Parametern der Durchführung einer Herzdruckmassage am Modell verglichen.

**Material und Methoden: **Die prospektiv, randomisierte Pilotstudie wurde im Sommersemester 2009 im Kölner Interprofessionellen SkillsLab und Simulationszentrum (KISS) an der Universität zu Köln durchgeführt. Die Probanden (Studierende der Humanmedizin des zweiten und dritten Fachsemesters) nahmen freiwillig an der Studie teil. Die Studierenden wurden in drei parallele Studiengruppen randomisiert, die nach jeweils einer der oben genannten Methoden unterrichtet wurden. Eine Woche sowie fünf bzw. sechs Monate nach Intervention wurden die Probanden in einer objektivierten strukturierten Einzel-Überprüfung bzgl. ihrer Reanimationsfertigkeiten geprüft. Die Ergebnisse der Gruppen wurden hinsichtlich der Parameter Kompressionsfrequenz, Kompressionstiefe, Anteil richtiger Kompressionen sowie Anzahl in einer Checkliste erreichter Items verglichen. Verglichen wurden außerdem Häufigkeitsverteilungen bezogen auf das Umsetzen oder Verfehlen damals geltender Leitlinienempfehlungen zur kardiopulmonalen Reanimation.

**Ergebnisse: **Die untersuchte Stichprobe umfasste 134 Probanden (68% weiblich; Alter: 22±4 Jahre; PEY: n=62; PMOD: n=31; STDM: n=41). Es bestand kein Unterschied zwischen den Gruppen bzgl. Alter, Geschlechterverteilung, Vorerfahrung oder zeitlichem Abstand zu einem vorherigen (Reanimations-) Kurs. Nur bei einem Endpunkt fand sich ein statistisch signifikanter Unterschied: Die mittlere Kompressionsfrequenz in der ersten Prüfung lag in Gruppe 1 (PEY) bei 99±17/Minute, in Gruppe 2 (PMOD) bei 101±16/Minute und in Gruppe 3 (STDM) bei 90±16/Minute (p=0,007 für Gruppe 3 vs. Gruppe 1 sowie Gruppe 3 vs. Gruppe 2 im Mann-Whitney-U-Test). Keiner der untersuchten Parameter unterschied sich zwischen den drei untersuchten Gruppen 5 bzw. 6 Monate nach Durchführung des Kurses.

**Schlussfolgerung:** Unsere Studie liefert keinen Hinweis auf wesentliche Unterschiede bei der Durchführung einer Herzdruckmassage durch Studierende im Rahmen der kardiopulmonalen Reanimation am Modell in Abhängigkeit von der Unterrichtsmethode (Peyton vs. „Non-Peyton“) bezogen auf den mittelfristigen Überprüfungszeitraum. Möglicherweise ist die Herzdruckmassage als Fertigkeit nicht komplex genug, um von der Vermittlung nach Peyton zu profitieren.

## 1. Introduction

The first human external chest compression was described in 1690 by Kouwenhoven et al. [1] and modified over time in terms of setting as well as compression rate, depth and point. It is an effective skill employed to maintain minimum circulation in order to provide organs intolerant against hypoxia with oxygen until the “Return of Spontaneous Circulation” (ROSC) occurs.

In Germany, there are approximately 123 sudden cardiac arrests per 100.000 people per year, in only 50-80 cases of which bystanders commence “Basic Life Support” (BLS) [2]. 48% of cardiac arrests occur in public but only in 23% of these do people start Cardiopulmonary Resuscitation (CPR) [3].

Fast diagnoses of cardiac arrest, immediate emergency calls and administration of good quality CPR are the keys to survival in and out of hospital [4].

It should therefore be of particular importance to teach these skills effectively. Practical (clinical) medical skills occupy an increasingly important role in German medical studies. From the amendment of the regulations for medical licensure [Ärztliche Approbationsordnung (ÄApprO)] in 2005 [5] up to the new “National competence based learning goal catalogue” (NKLM), [Nationaler Kompetenzbasierter Lernzielkatalog Medizin (NKLM)], all of these emphasize the importance of practical clinical skills [6].

There is a need for simple teaching methods every teacher can adopt, which are accepted by the students and provide a sustainable outcome. A widespread methodical approach is Peyton’s 4-Steps-Approach [7], [8]. The approach by R. Peyton [9] to teaching practical clinical skills consists of four steps:

1. **Demonstration: **The teacher performs the skill in real time without comment. This step is taken to provide a benchmark.
2. **Deconstruction:** The teacher performs every step slowly with an added explanation. The skill should be divided into smaller subsections.
3. **Comprehension:** The student describes every step of the skill whereupon the teacher performs on instruction. The description and execution do not occur simultaneously.
4. **Execution: **The student simultaneously narrates and executes step by step.

Peyton’s approach combines multiple aspects of learning theory. The learning in Steps 1 and 2 is based on a social-cognitive approach to learning theory, that of model-learning according to Banduras [10], whereas Step 4, the actual implementation and training of the procedure up to its successful application, is associated with the behaviourist learning theory.

According to Jawhari et al. [11], the third step of Peyton’s approach is crucial: „The perceptually processed information (Step 1 & Step 2) must be actively manipulated in the working memory in Step 3 to be transferred into the long-term memory“. According to Krautter et al. [12], the description of the procedure without simultaneous administration produces a mental correlate of the procedural motions, which leads to more efficient motor learning and better reproduction.

From a constructivist view of learning theory (as in the sense of constructivist pedagogy according to Reich 1997 [13]) Peyton’s approach can be described as the endeavour to create a constructive “place of furthest reaching own world invention” (ibid. 266).

Consequently, a combination of all four steps would be necessary in order to achieve success in learning in the sense of a well-established self-construction.

Thus, our study was based upon two hypotheses:

1. The execution of all four steps according to Peyton is superior to modifications that omit one or more of the steps in terms of medium-term learning success.
2. Step 3 of Peyton’s approach is crucial and the sole omission of this step diminishes the generated learning success.

Based on this we compared the learning success of medical students taught a) according to Peyton (PEY^1^; Steps 1-4), b) under omission of Step 3 (PMOD^1^) or c) only with Steps 2 & 4 according to Peyton (STDM^1^), once a week later and again 5 to 6 months later. The learning success was determined through the practical test of an external chest compression in the context of cardiopulmonary resuscitation on a model.

The 4-Step method according to Peyton as an approach to the instruction of practical skills has already been empirically researched. Some of the surveys conveyed that students taught with the help of this method profited from their instruction in comparison with other groups [11], [12], [14], [15], [16]. Other surveys found no advantages to the Peyton method [17], [18], [19]. One study compared the 4-Step method with the 2-Step standard method “See one, do one” in the instruction of cardiopulmonary resuscitation (CPR), just as we did for this very paper, that study however only conducted research into immediate short-term effects after course completion [20]. Despite the generally contradictory and, with respect to CPR, insufficient existence of surveys, Peyton’s 4-Step method has been propagated for the instruction of cardiopulmonary resuscitation by Bullock [21] since the beginning of the twenty-first century, and recommended by the European Resuscitation Council (ERC) until 2015 [22], [23], [24].

For these reasons (and regarding Best Evidence Medical Education, BEME [25], [26]), we have researched the instruction of cardiopulmonary resuscitation by means of the Peyton method compared to modified means of instruction for this particular study.

## 2. Material and methods

### 2.1. Study design

This prospective and randomised pilot study with three parallel study groups took place during the summer semester of 2009 as part of the first aid course at the Cologne Interprofessional SkillsLab and Simulation Centre (KISS) of the University of Cologne. 

To this end, the at that point current guidelines of resuscitation of 2005 [27], [28] were taught and appropriately tested. Consequently, there are discrepancies from the now current guidelines of the ERC concerning the resuscitation data.

#### 2.1.1. Test subjects

The test subjects of this study were medical students in their second and third semester of graduate school. Participation in the study was voluntary and all subjects enrolled in the course were randomised. The contents and time frame of the course were identical to those of the rescue organisations, but our course required the successful completion of a practical test. The practical instruction in resuscitation alone took up 90 minutes. Tutors of the SkillsLab employed a variety of instructional methods in compliance with the study protocol. The course size was limited to a maximum of 16 participants (Median=13; at a maximum of 16 participants and a minimum of 9 participants).

**Figure 1:**
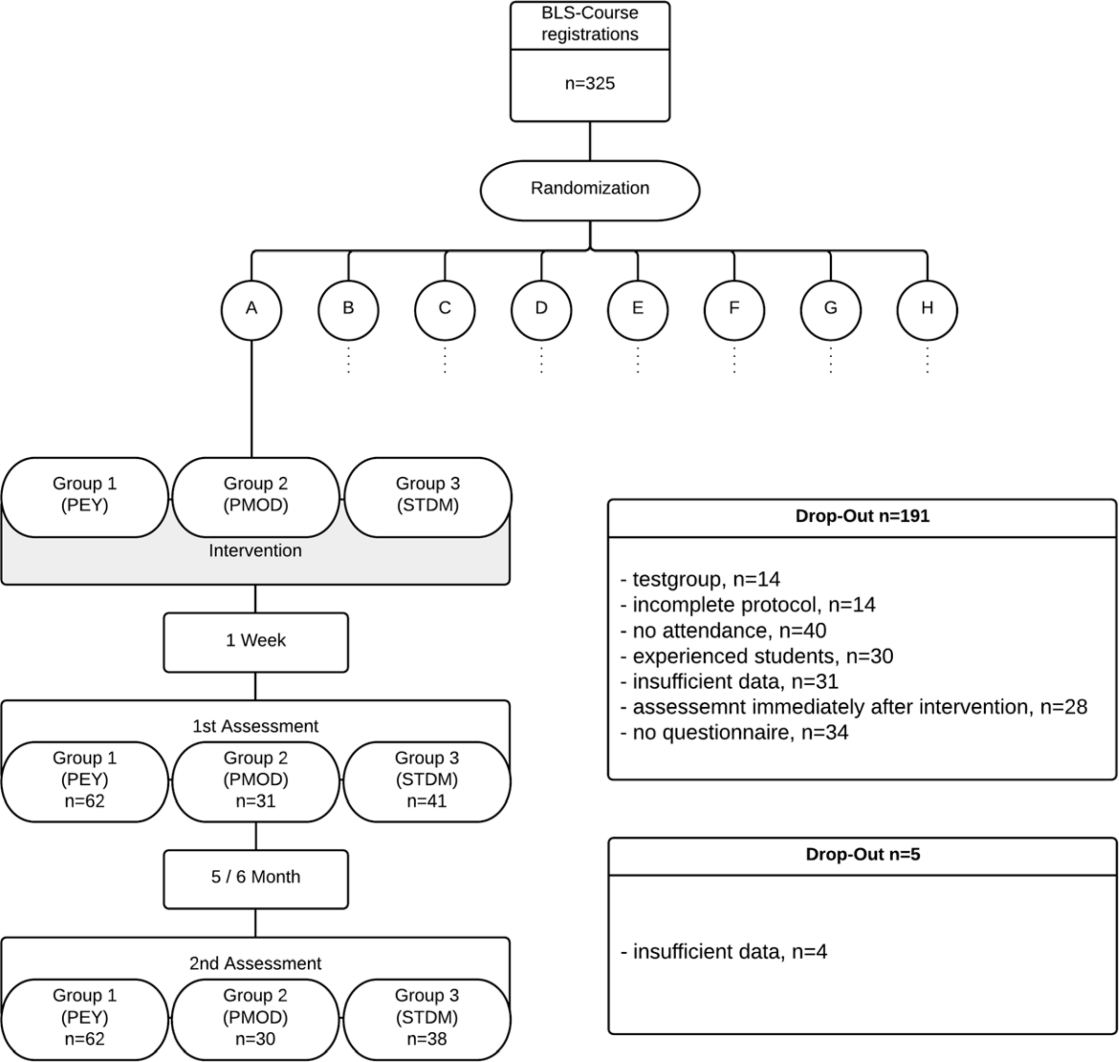
Procedure and Drop-Out

The evaluation only included participants present on the date of the intervention and on the day of the test a week later, as well as having filled out and submitted a questionnaire with epidemiologic data. Test subjects with prior experience^2^ were also excluded. Further criteria for exclusion are depicted in Figure 1 (Fig. 1).

##### 2.1.2. Tutors/ examiners

The course tutors were student employees of the SkillsLab in higher clinical semesters of graduate school, who were also experienced in resuscitation due to prior work in emergency medical services before or during their studies. Being tutors in other courses, they were also trained in the instruction of practical skills, as well as having been prepared for the study in a separate training seminar. The tutors were also implemented as examiners, but only for groups they hadn’t tutored beforehand. 

##### 2.1.3. Blinding, data privacy, ethics

All subjects enrolled in the course agreed to pseudonymous collection of data as well as anonymous data processing and evaluation in writing^3^. The participants were given no information concerning the aim of the study, they were only informed about their participation in it. The tutors were not blinded. The emergency education in the course of medical studies at the University of Cologne prescribes annual emergency trainings. Each seminar begins with a revision of known skills learnt beforehand and continues with the instruction of new contents. This way, the students successively further their personal emergency competence with every year. The external chest compression is a firm part of every emergency course. By way of this longitudinal emergency training, we considered no disadvantages for participants of our study regarding the acquisition of personal emergency competence. Potential shortcomings can be compensated during the remainder of their studies.

#### 2.2. Randomisation

The subjects were divided into eight groups (A-H) through random numbers (generated by SPSS) with further randomised divisions in each of the eight groups into three instruction groups (Group 1 (PEY), Group 2 (PMOD) and Group 3 (STDM)). The first division clarified the period of instruction: groups A-D were tutored in the first four weeks of semester, groups E-H in weeks five to eight^4^. The second randomisation determined the instruction/ intervention group. The assignment of tutors to different instruction groups was also randomised.

##### 2.2.1. Course of events

In accordance with the fixed study protocol, the groups were tutored and assessed through an objective, structured evaluation regarding their resuscitation competence one week after intervention.

**First assessment (after 1 week)**

In this setting, the students were given one minute to read through the description of a situation containing the tasks and contextual conditions (one-helper method). Afterwards, the subjects were given five minutes to complete their tasks:

1. Assessment of the necessity for resuscitation
2. Begin and administration of resuscitation

The students were evaluated by an examiner using a (binary) checklist and the relevant performance data of the resuscitation phantom was collected (Laerdal, Resusci Anne Advance Skill Reporter^TM^).

**Figure 2:**
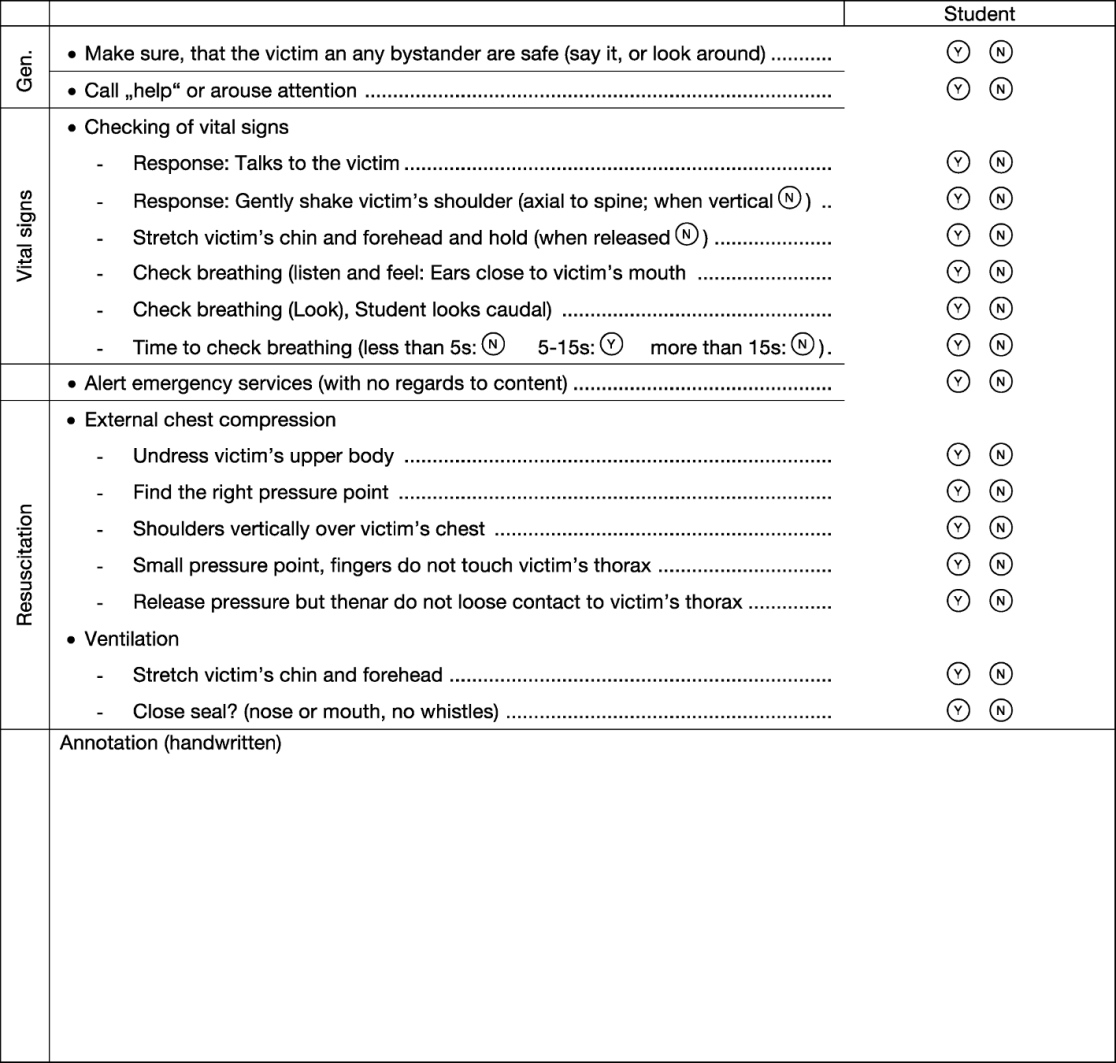
Checklist items first Assessment (16 items)

The checklist contained 16 dichotomous items (see Figure 2 (Fig. 2)), all of which were derived from the resuscitation guidelines of 2005 [27]. The completion of the test occurred after more than five full resuscitation cycles or after two minutes of continuous external chest compression, if the task was the administration of “compression-only” resuscitation.

**Second assessment (after 5-6 months)**

**Figure 3:**
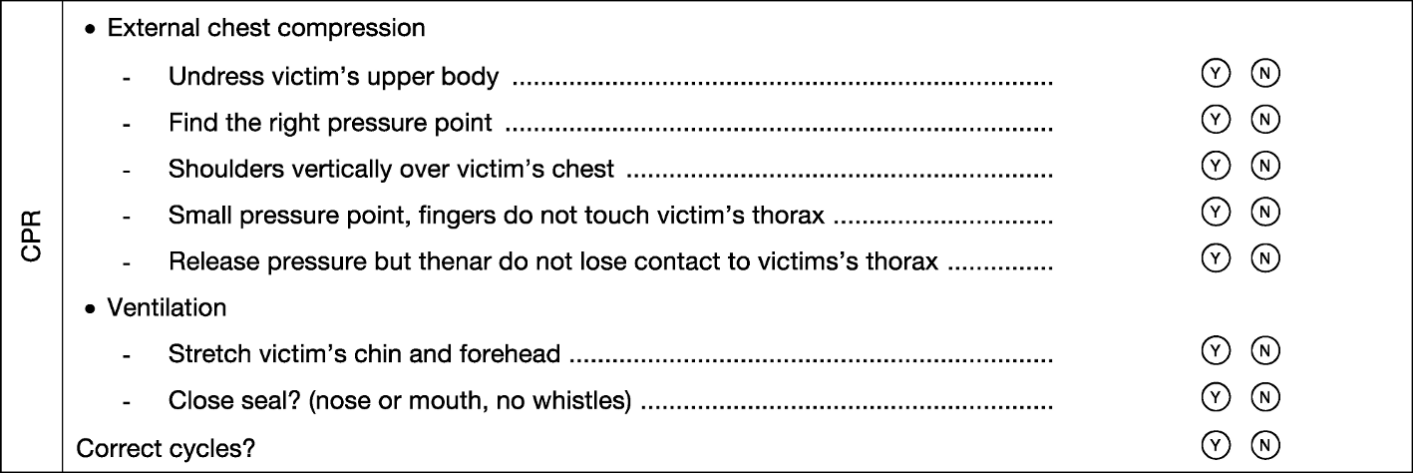
Checklist items second Assessment (8 items)

Five months (groups E-H) and accordingly six months (groups A-D) after course completion, the students were once again assessed through an objectively structured practical test respective of the final test of the first aid course. The data collected concerning resuscitation was evaluated in this study. For reasons pertaining to the right of inspection, a different, already existing checklist containing eight items was used (see Figure 3 (Fig. 3)). The resuscitation was performed under the same conditions as in the first assessment.

The collection of performance data regarding the phantom occurred using the same resuscitation model. The students had been informed about the test, were familiar with the contents of the course and were able to prepare for the test with the help of a resuscitation phantom. 

#### 2.3.Teaching methods

For this study, the steps were modified as follows. 

##### 2.3.1.Group 1 (PEY): 4-Step method according to Peyton

Since the original approach according to Peyton was conceived for one-on-one assistance in the operating theatre, we respectively appropriated the comprehension step (Step 3) to the group setting.

- Step 1 (Demonstration) and 2 (Deconstruction): Unaltered.
- Step 3 (Comprehension):
  1. One student describes the course of action, the tutor then executes this course while the rest of the group observes.
  2. Groups of three students each were formed around a resuscitation phantom. One student describes the course of action, another executes it while the third remaining student takes on the role of the observer. After the completion of the administration, the roles are switched in such a way that every subject has to have executed “Step 3 (Comprehension)”. The course tutor “monitors” group formation and steps in to correct mistakes if need be.
- Step 4 (Execution): Unaltered, groups of three practice resuscitation on a phantom.

##### 2.3.2. Group 2 (PMOD): Omission of Step 3 according to Peyton

- Step 1 (Demonstration) und Step 2 (Deconstruction): Unaltered.
- Step 3 (Comprehension): Omitted.
- Step 4 (Execution): Groups of three practice resuscitation on a phantom without the division of roles described in 2.3.1.

##### 2.3.3. Group 3 (STDM): Omission of Steps 1 and 3

- Step 1 (Demonstration): Omitted.
- Step 2 (Deconstruction): Unaltered.
- Step 3 (Comprehension): Omitted.
- Step 4 (Execution): Groups of three practice resuscitation on a phantom without the division of roles described in 2.3.1.

This complies with the so-called classic instruction “See one, do one”. The tutor explains and demonstrates the resuscitation (Step 2), after which all questions and uncertainties are clarified and practice on a resuscitation phantom in groups of three follows.

##### 2.3.4. Evaluation, resources, data work flow

The checklist items were collected on paper, digitalised using OMR-Office® Version 5 of the company Remark® and ported using Excel® versions 12 and 14 for Mac OSX of the company Microsoft. The CPR data was collected using the resuscitation model Resusci Anne Advanced Skill Reporter® of the company Laerdal®, assigned to the subjects per hardcopy and then digitalised. For statistical analysis we employed SPSS® versions 20 and 22 for Mac OSX of the company IBM®. With regards to the quality of resuscitation, we considered the parameters depicted in Table 1 (Tab. 1), on the one hand as actual measured value for direct comparison, but also categorised into dichotomous values (correct/ false). The basis of categorisation was provided by the resuscitation guidelines of 2005 [27] as well as the publications of Kern et al. 1992 [29] and Abella et al. 2005 [30] for the determination of the permitted average compression rate of 90-110 spm. The same targeted frequency range was later also employed by Sopka et al. [23] and Jenko et al. [20]. We arbitrarily determined a limit of 60% in the sense of a fail for the number of “correct compressions^5^” and checklist items.

#### 2.4. Statistical methods

The data was processed and analysed via SPSS (IBM® SPSS® version 22, Mac OSX). The testing of the data for normal distribution was performed through visual inspection via histogram and Q-Q-Plot, as well as mathematically with the aid of the Shapiro-Wilks-Test. Testing for significant differences in the epidemiologic data of the groups was calculated via Chi-Square-Test and Fischer’s Exact Test. Because not all the data was normally distributed, testing for differences in age distribution and CPR data was calculated according to Kruskal-Wallis. The Chi-Square-Test was used again for the comparison of categorised data. The level of significance was reduced to 0.0125 according to Bonferroni due to the comparison of four features. In order to simplify evaluation, the assumption was made that no interdependencies existed between the results of single subjects of a group. Therefore, all statements on statistical significance must be understood in the exploratory sense. Consistent values are given as arithmetic mean±standard deviation (MW±SD). 

## 3. Findings

For a tabular summary see Table 1 (Tab. 1), Table 2 (Tab. 2), Table 3 (Tab. 3), Table 4 (Tab. 4) and Table 5 (Tab. 5).

### 3.1. First Assessment, one week after intervention:

The analysed sample consists of 134 subjects (68% female; age 22±4 years; PEY: n=62; PMOD: n=31; STDM: n=41). There was no significant difference between the groups in terms of gender distribution (p= 0,887 [χ^2^-Test]), pre-existing CPR experience (p=0,790 [Fischer’s Exact Test]), time of last CPR course (p=0,582 [χ^2^-Test]) and number of compressions during CPR (p=0,064 [Kruskal-Wallis], see Table 2 (Tab. 2) and Table 3 (Tab. 3)). For exclusion criteria see Figure 1 (Fig. 1).

#### 3.1.1. Compression rate

We calculated a mean compression rate in the first assessment for Group 1 (PEY): 99±17/minute, Group 2 (PMOD): 101±16/minute and Group 3 (STDM): 90±16/minute (p=0,007 for Group 3 vs. Group 1, as well as Group 3 vs. Group 2 in Mann-Whitney-U-Test). The percentage of subjects with the correct compression rate (defined a priori as 90-110 bpm) was 29 subjects for Group 1 (PEY) (47%), 13 subjects for Group 2 (PMOD) (42%) and 14 subjects for Group 3 (STDM) (34%).

There was no significant difference between the groups, p=0,451 [χ^2^-Test].

##### 3.1.2. Compression depth

There was no significant difference in mean compression depth between the three groups: Group 1 (PEY): 36±10 mm; Group 2 (PMOD): 38±8 mm, Group 3 (STDM): 38±11 mm (p=0,572 [Kruskal-Wallis]). The comparison of fractions with correct compression depth (defined a priori as ranging from 40-50 mm) showed the following: Group 1 (PEY) with 25 subjects (40%), Group 2 (PMOD) with 15 subjects (48%) and Group 3 (STDM) with 14 subjects (34%) within the correct range of compression depth. There was no significant difference between the groups, p= 0,457 [χ^2^-Test].

##### 3.1.3. Correct Compression

When comparing “correct compressions”^5^ to all compressions in percentages, there is no visible significant difference between the groups (p=0,417 [Kruskal-Wallis]): Group 1 (PEY): 38%±34; Group 2 (PMOD): 44%±34; Group 3 (STDM): 43%±32.

When comparing the number of subjects with at least 60% “correct compressions”, we found 18 subjects in Group 1(PEY) (29%), 13 subjects in Group 2 (PMOD) (42%) and 14 subjects in Group 3 (STDM) (34%) with 60% or more correct compressions (p=0,470 [χ^2^-Test]). 

##### 3.1.4. Checklist items

When comparing the number of correct checklist items (at a maximum of 16), we found no significant difference between the groups (Group 1 (PEY): 13±2; Group 2 (PMOD): 13±2; Group 3 (STDM): 13±2), p=0,487 [Kruskal-Wallis]). When comparing the number of subjects with at least 60% correct checklist items, we found no significant difference either (p=0,479, [χ^2^-Test]). Group 1 (PEY) had 58 (94%) subjects, Group 2 (PMOD) had 31 (100%) subjects and Group 3 (STDM) had 39 (95%) subjects with 60% correct checklist items or more.

#### 3.2. Second assessment, five/six months after intervention

Compared to the first assessment, a loss of 4 subjects occurred because of insufficient data.

##### 3.2.1. Compression rate

The arithmetic mean of the average compression rate was 112±12/minute in Group 1 (PEY), 113±13/minute in Group 2 (PMOD) and 108±15/minute in Group 3 (STDM) (p=0,600 in the Kruskal-Wallis-Test). When comparing the percentage of subjects with a mean compression rate within the a priori determined range of 90-110/minute, there were 27 subjects in Group 1 (PEY) (44%), 12 subjects in Group 2 (PMOD) (40%), and 14 subjects in Group 3 (STDM) (37%). There was no evidence of significant difference between the groups (p=0,801 [χ^2^-Test]).

##### 3.2.2. Compression depth

We could not detect any significant difference concerning the average compression depth when comparing the individual groups, (p=0,942 in the 'Kruskal-Wallis-Test'). We measured an average compression depth of 42±8mm for Group 1 (PEY), 42±7mm for Group 2 (PMOD) and 42±8mm for Group 3 (STDM).

Comparing the number of students with an average compression depth within the a priori determined target range from 40-50mm, we counted 33 subjects for Group 1 (PEY) (53%), 13 subjects for Group 2 (PMOD) (43%) and 19 subjects for Group 3 (STDM) (50%) (p= 0,669, [χ^2^-Test]).

##### 3.2.3. Correct compressions

Comparing the percentages of “correct compressions”^5^, we found 49%±32 for Group 1 (PEY), 53%±30 for Group 2 (PMOD) and 48%±31 for Group 3 (STDM) of correct compressions (p=0,840, in the “Kruskal-Wallis-Test”).

Comparing the groups with regard to the number of subjects with at least 60% correct compressions, we found 27 subjects in Group 1 (PEY) (44%), 14 subjects in Group 2 (PMOD) (47%) and 17 subjects in Group 3 (STDM) (45%) (p=0,973, [χ^2^-Test]).

##### 3.2.4. Checklist items

The following mean values were produced for the different groups by the comparison of their number of correct checklist items (at max. 8): Group 1 (PEY) 7±1; Group 2 (PMOD) 8±1 and Group 3 (STDM): 8±1 (p=0,824, Kruskal-Wallis-Test).

We could not detect a significant difference between the groups within the comparison of the fraction of subjects with at least 60% correct checklist items (p=1,000 [χ^2^-Test]). In Group 1 (PEY), this fraction was 61 subjects (98%), in Group 2 (PMOD) it was 30 (100%) and in Group 3 (STDM) it was 38 (100%).

## 4. Discussion

In this study, we compared three different methods of practical instruction of basic life support with respect to medium-term learning achievement of resuscitation performance. This aspect is of importance because of the necessity of using sustainable instruction methods precisely for the instruction of basic life support. The accessibility of CPR skills decreases over time [31] and corresponds to roughly the level before a course after one to two years [32]. Jenko et al. [20] were able to illustrate the absence of advantages of the instruction of cardiopulmonary resuscitation via Peyton’s 4-Step method compared to the 2-Step standard model (“See one, do one”) concerning the short-term learning achievement (immediately after course completion). A further study, published as an abstract, found no difference between groups taught using Peyton’s method and those taught using the 2-Step method after a period of three months [19]. However, the aim of that course was the administration of the recovery position, which is why those findings can only be compared to ours to a certain extent.

In the collective we researched, there was only one significant difference, namely the mean compression rate in the first assessment (one week after intervention). The subjects in Group 3 (STDM) instructed according to the “See one, do one” approach we call the standard model (Peyton’s Steps 2 and 4), were significantly slower in the first assessment one week after the intervention than the subjects in the other groups (PEY: Steps 1-4; PMOD: Steps 1, 2, and 4 (see Table 4 (Tab. 4))). The average compression rate in Group 3 (STDM) was still within the limits prescribed by the then current guidelines for resuscitation. There was no significant difference when comparing the percentages of subjects with the correct compression rate. Consequently, the practical relevance of the observed difference in mean compression rate dependent on the teaching method is improbable. The limitation is set higher in the recently released new guidelines for resuscitation by the ERC [33]: 100-120 spm. Nonetheless, these were not the basis of the course and therefore unknown by the students. Further studies must show which factors influence the learning of the correct compression rate. Instruction in conjunction with music, for example, supposedly leads to a positive effect on learning according to Hafner et al. [34].

The significant difference in mean compression rate was not evident in the assessment 5 to 6 months later. We attribute this most likely to a training and practice effect, due to the subjects’ knowledge of a second assessment and their subsequent chance at preparing themselves specifically for it. However, this could also be a reason for our inability to measure differences in the medium term, since deficits stemming from the instruction method could have been compensated through targeted training. The preparation time was not separately recorded, hence we do not know of the existence of differences in preparation time between different groups. The randomisation of the participants should ensure the rough equivalence of this “risk” in all groups. Apart from a systematic recording, we have no reason to believe that more training material (reanimation phantoms) was taken advantage of before the second assessment than in previous semesters, nor that corresponding training rooms were more highly frequented (loan of material and room keys occurs solely through our SkillsLab). The students were not informed about which group they were assigned to. It is, however, a safe assumption that the students exchanged details of the course contents amongst themselves and thus realised there were differences in instruction. This means we can only talk of blinding to a certain extent, which in turn means we cannot rule out the possibility of distortion. Whether this had an impact on the measurable performance of resuscitation, specifically on the compression rate, is questionable. A structured registration of preparation time, frequency and partners should therefore occur in a follow-up study. The division of subjects into two large subcategories (first and subsequent four weeks of semester) was necessary due to the prescribed schedule intending these time periods for the first aid course. The randomisation should have excluded the possibility of distortion in this case. 

We appointed student employees of the SkillsLab as tutors, who had been trained beforehand. The equal suitability of trained students as tutors to that of professional staff has been illustrated by Tolsgaard et al. [35], among others. The final analysable number of cases was significantly reduced due to an unexpectedly high drop-out rate. With respect to our recorded prevalence for the correct compression rate of Group 1 (PEY) and Group 3 (STDM), 294 subjects per group based on a power of 80% and α=0.05 would have to be researched in a new study. For the prevalence of the correct compression depth, the amount would be 985 subjects. Our study is therefore classified as underpowered and can only be considered a pilot study. It is possible that our transfer of Peyton’s 4-Step method from a one-on-one setting into a group setting decreased its effectiveness. Roughly only two thirds of the students in Group 1 (PEY) followed the correct order of Peyton’s 4-Step method, i.e. the verbalisation of the action prior to its execution. A third performed the action first contingent upon modification. Although this occurred following the instruction of the subject who was executing Step 3 rather than their own intention, the originally intended order was nevertheless not followed. Nikendei et al. also applied Peyton’s approach to a group context [36]. In their case, the originally intended order of steps was correctly followed for all students. Further studies must show, whether the order of Peyton’s steps has crucial impact on the result, or whether the third step, for example, still has an effect after the first motor performance of the action. 

In conclusion, we were unable to prove a superiority of Peyton’s 4-Steps-Approach (PEY) compared to the version modified through “omission of Step 3” (PMOD), nor compared to the “See one, do one” method (STDM) in our study with the limitations specified above. In the recently published new guidelines for resuscitation of the ERC, the recommendation for the application of the 4-Step method is revoked [31]. This retraction is, however, based on studies that focused their research on the instruction of other skills^6^ than cardiopulmonary resuscitation [17], [18].

In our opinion, the transfer of these study results onto the instruction of CPR is nonetheless not possible. Peyton’s 4-Steps-Approach was originally described as a way of acquiring and practicing complex clinical skills (specifically practical operative skills) [9]. There are studies that corroborate the effectiveness of Peyton’s method. Gradl et al. [16] illustrated the superiority of Peyton’s method in the publication of an abstract on the instruction of complex manual-therapeutic skills, wherein 100 dichotomous items were observed. Krautter et al. [12] were able to illustrate the superiority of results gained by the instruction of a central venous catheter (CVC) insertion via the complete 4-Step method according to Peyton, as a complex clinical skill encompassing 39 procedural treatment instructions, compared to only using parts of the 4-Step method. Contrary to these results, Bjørnshave et al. [19] showed in a published abstract that subjects who were taught the maneuvering of an unconscious person into the recovery position in 8 procedural steps did not profit from the 4-Step method. Greif et al. [17] also illustrated this with respect to the instruction of an emergency cricothyroidotomy (percutaneous needle-puncture cricothyroidotomy) with 10 procedural steps, as well as Orde et al. [18] for the instruction of the larynx mask insertion with 9 procedural steps. Peyton’s 4-Steps-Approach seems not to offer a measurable advantage in the context of less complex clinical (emergency) skills. Over the years, the complexity of basic life support has been steadily reduced so as to be easily accessible for a wide range of learners and quickly accessible in case of an emergency. This can be exemplified by the finding of a pressure point: In the resuscitation guidelines of 2000 [37], the finding of the correct pressure point alone consists of six successive instructions, five years later the same procedure consists of only one step [27]. Currently, the entirety of resuscitation is condensed into three words by the “Save a life” campaign of the professional organisation of German anaesthesiologists and the German Society of Anaesthesiology and Intensive Care Medicine (DGAI): “Check, call, press” [https://www.einlebenretten.de/].

Our study contained 19 procedural steps (16 dichotomous items an 3 resuscitation parameters, see Figure 2 (Fig. 2) and Table 4 (Tab. 4)). It is therefore conceivable, that the reduction in complexity renders Peyton’s 4-Steps-Approach less effective in higher selected subject collectives (such as medical students). The approach would nonetheless be interesting regarding wider education (medical laypersons). Krautter et al. 2011 [14] also illustrated the superiority of Peyton’s approach on another level in terms of the instruction of gastric-tube insertion: Procedurally compared (through checklists, 13 dichotomous items) the 4-Step method was not superior to the classic instruction method, it did however display advantages with respect to doctor-patient communication, professional manner and the speed of execution of the activity. This aspect was also shown by Lund et al. [15] for the instruction of an IV cannulation, although the intervention group (4-Step method) in this study was also superior to the control group (“See one, do one”) in procedural comparison (25 dichotomous items). The dimension of the Peyton method that transcends procedural comparison was not considered in our study, since communicative and hygienic skills as well as professional manner are not of utmost importance in basic life support. This aspect does, however, become important when it comes to the instruction of advanced resuscitation skills (Advanced Life Support, ALS), where team communication and (leading) role assignment are crucial.

Further studies with larger numbers of cases should clarify, whether following the order of Peyton’s steps is obligatory, whether the choice of teaching method has an impact on the participants’ motivation, whether the assimilation of competence and sense of mastery can be achieved and whether the teaching method should be adjusted to the target audience. Furthermore, the suitability of the 4-Step method according to Peyton as a tool for inexperienced tutors to gain structure and security in the instruction of practical skills needs to be clarified, as well as the optimal subject group size for this instructional approach. 

More creativity and courage to try out new teaching methods should be shown in the search for teaching methods for basic life support in tutored courses, each of which should of course be scientifically accompanied and evaluated with respect to Best Evidence Medical Education (BEME). Peyton’s 4-Steps-Approach should be questioned in the sense of a “dogma” [38], which has already occurred through the recent release of the new resuscitation guidelines of the ERC, it should nevertheless not be completely taken out of the instructional repertoire for cardiopulmonary resuscitation.

## Notes

^1^ PEY=Peyton; PMOD=Peyton modified; STDM=standard model

^2^ Prior experience: first aid course at some point during the last two years, apprenticeship as paramedic, physiotherapist, nurse, in emergency rescue services

^3^ In the model course of studies in Cologne, students globally accept the personalised, pseudonymous and/ or anonymous collection and processing, but never the personalised publication, of their personal data for research purposes.

^4 ^These time periods were reserved for the first aid course in the course of studies and were not freely changeable by us.

^5^ “Correct compressions” or “Compressions without faults” are compressions with the correct pressure point, the correct position and with complete decompression after each single compression.

^6^ Larynx mask insertion [18] and percutaneous needle-puncture cricothyroidotomy [17]

## Acknowledgements

Many thanks go to the numerous students working at our SkillsLab, who supported this study and made it possible. Special thanks in particular go to: Elisabeth Sauer, Katharina Albrecht, Carsten Wessels, Patrick Lang, Daniel Weber, Traugott Gruppe and David Schwarz.

## Competing interests

The authors declare that they have no competing interests.

## Figures and Tables

**Table 1:**
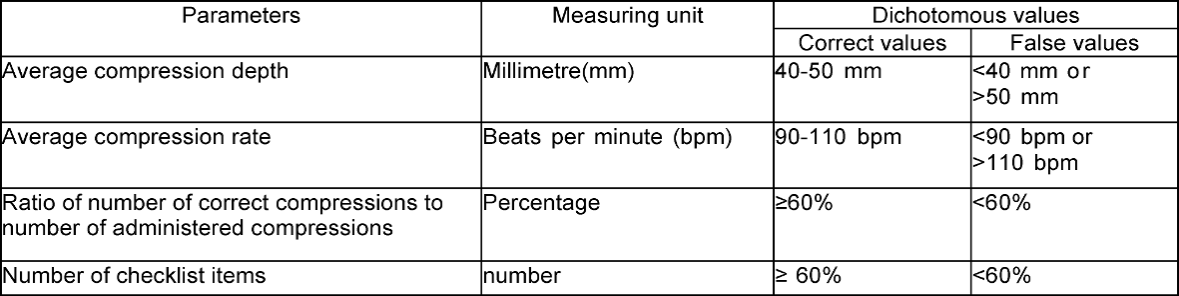
CPR target value

**Table 2:**
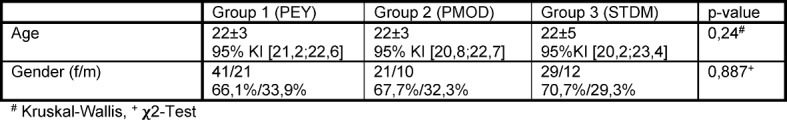
Epidemiological data 1

**Table 3:**
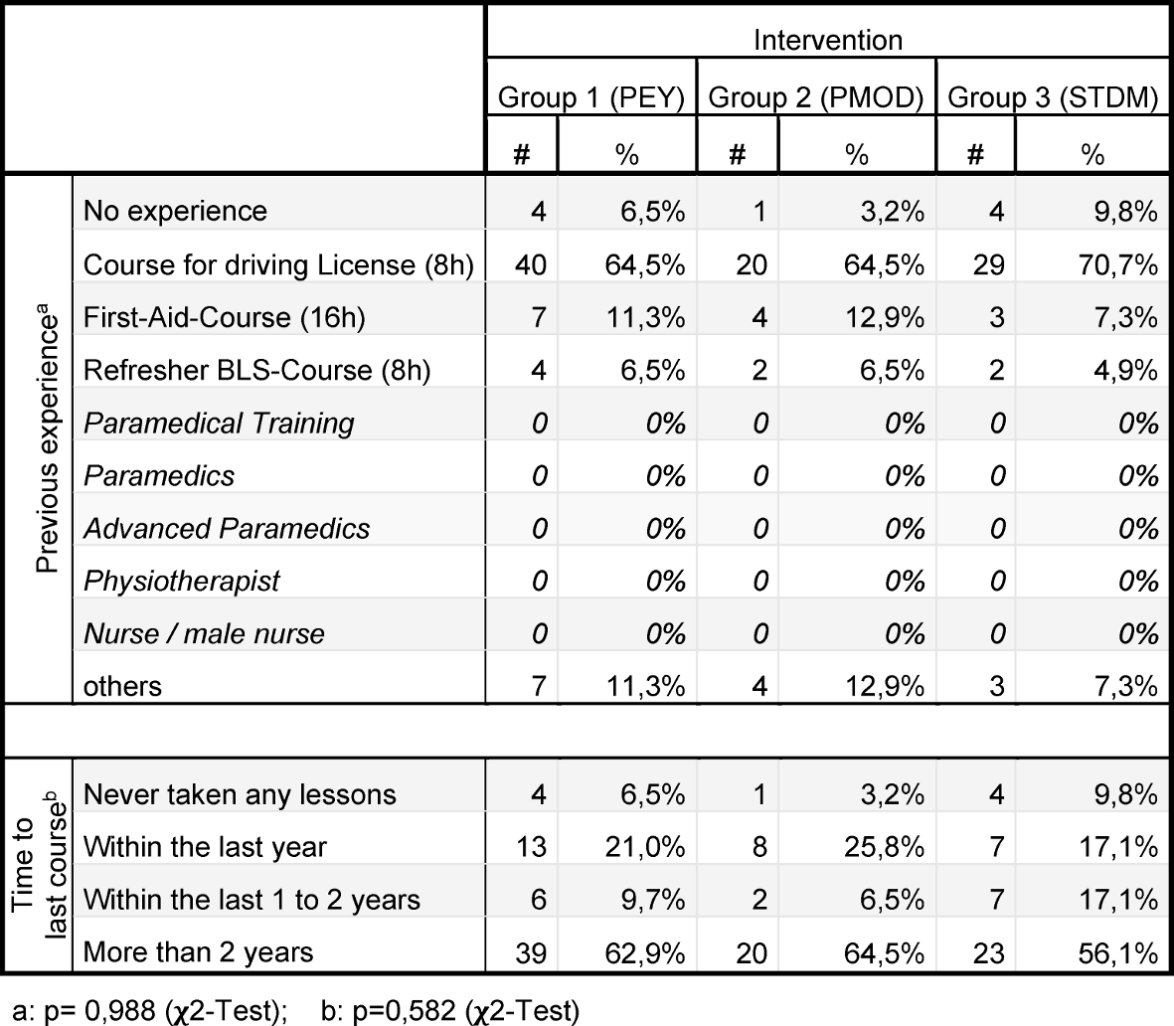
Epidemiological data 2: Previous experience, time of last course

**Table 4:**
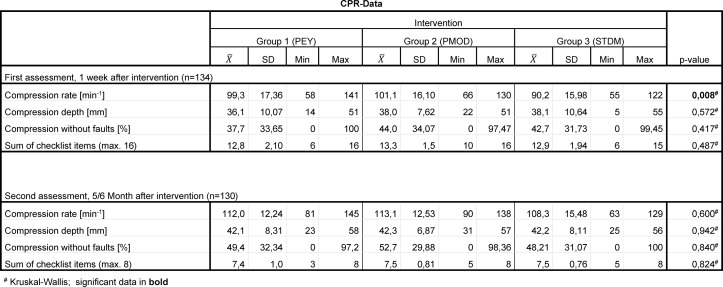
CPR-Data

**Table 5:**
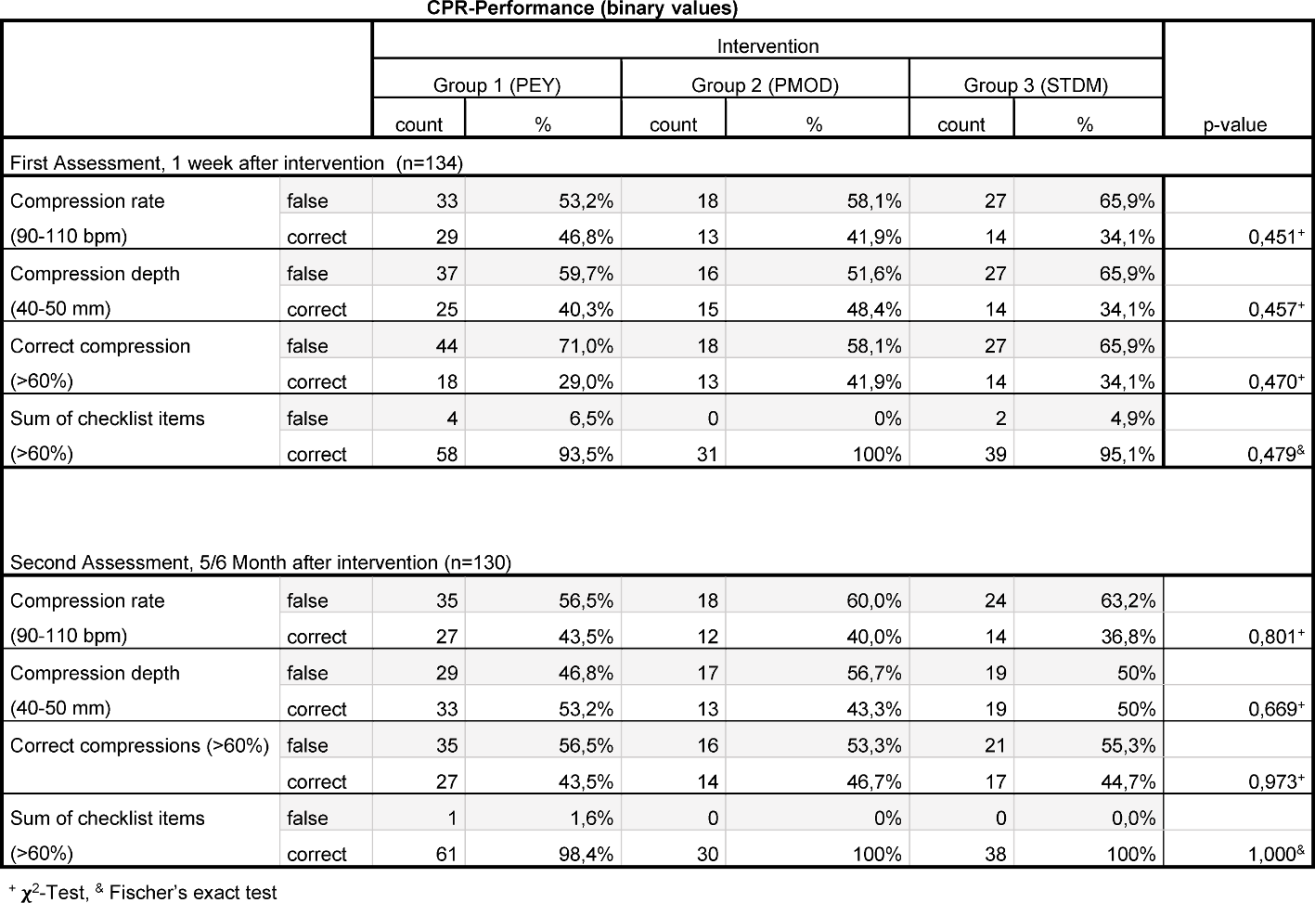
Binary CPR-Data
